# Facile preparation of silver based radiosensitizers via biomineralization method for enhanced in vivo breast cancer radiotherapy

**DOI:** 10.1038/s41598-023-40763-9

**Published:** 2023-09-13

**Authors:** Mohammadreza Ghaffarlou, Ali Mohammadi, Navid Mousazadeh, Marziyeh Salehiabar, Yahya Kalantari, Jalil Charmi, Murat Barsbay, Yavuz Nuri Ertas, Hossein Danafar, Hamed Rezaeejam, Hamed Nosrati, Siamak Javani

**Affiliations:** 1 Department of Chemistry, Hacettepe University, Beytepe, Ankara, 06800 Turkey; 2 Zanjan Pharmaceutical Biotechnology Research Center, Zanjan University of Medical Sciences, Zanjan, Iran; 3 ERNAM-Nanotechnology Research and Application Center, Erciyes University, Kayseri, 38039 Turkey; 4 Department of Biomedical Engineering, Erciyes University, Kayseri, 38039 Turkey; 5 Department of Radiology Technology, School of Allied Medical Sciences, Zanjan University of Medical Sciences, Zanjan, 45139-56184 Iran; 6 Medical Cellular and Molecular Research Center, Golestan University of Medical Sciences, Gorgan, Iran; 7 School of Advanced Technologies in Medicine, Golestan University of Medical Sciences, Gorgan, Iran

**Keywords:** Bioinorganic chemistry, Biopolymers in vivo, Nanoscale biophysics

## Abstract

To solve the traditional radiotherapy obstacles, and also to enhance the radiation therapy efficacy various radiosensitizers have been developed. Radiosensitizers are promising agents that under X-ray irradiation enhance injury to tumor tissue by accelerating DNA damage. In this report, silver-silver sulfide nanoparticles (Ag-Ag_2_S NPs) were synthesized via a facile, one-pot and environmentally friendly biomineralization method. Ag-Ag_2_S was coated with bovine serum albumin (BSA) in situ and applied as an X-ray sensitizer to enhance the efficiency of radiotherapy. Also, folic acid (FA) was conjugated to Ag-Ag_2_S@BSA to impart active targeting capability to the final formulation (Ag-Ag_2_S@BSA-FA). Prepared NPs were characterized by transmission electron microscopes (TEM), scanning electron microscope (SEM), dynamic light scattering (DLS), ultraviolet–visible spectroscopy (UV–Vis), X-ray diffraction analysis (XRD), and X-ray photoelectron spectroscopy (XPS) techniques. Results show that most of the NPs have well-defined uniform Janus structures. The biocompatibility of the NPs was then evaluated both in vitro and in vivo. A series of in vitro assays were performed on 4T1 cancer cells to evaluate the therapeutic efficacy of the designed NPs. In addition, the radio-enhancing ability of the NPs was tested on the 4T1 breast cancer murine model. MTT, live and dead cell staining, apoptosis, ROS generation, and clonogenic in vitro assays demonstrated the efficacy of NPs as radiosensitizers in radiotherapy. In vivo results as well as H&E staining tumor tissues confirmed tumor destruction in the group that received Ag-Ag_2_S@BSA-FA NPs and exposed to X-ray. The results showed that prepared tumor-targeted Ag-Ag_2_S@BSA-FA NPs could be potential candidates as radiosensitizers for enhanced radiotherapy.

## Introduction

Cancer is an uncontrolled process of abnormal cell growth that inhibits apoptosis messages in cells, causing serious disease and highly noticeable mortality, almost 10 million deaths worldwide in 2020. Breast cancer affects one out of four women and is the cause of one out of every six cancer deaths and ranks first in incidence in the majority of countries (159 out of 185)^1–3^. Cancer has traditionally been treated with surgery and chemotherapy. Radiation therapy (RT) is a branch of oncology based on radiation therapy that can be used alone or in combination with surgery and chemotherapy to achieve local control of breast cancer. RT often uses high-energy radiation, such as X-rays or gamma rays to ionize atoms or molecules in order to directly or indirectly damage DNA blocks in cancer cells or to destroy the tumor, which is employed in at least 50% of all solid malignancies, and extends the life expectancy of cancer patients^4,5^. Acute or late toxicity, such as edema, fibrosis, and telangiectasia, can be seen in normal tissue during radiation treatment, which poses significant barriers to increasing the radiation dose. Therefore, decreasing the dosage of RT may be an appropriate target to eradicate the aforementioned problems. To address this issue, different radiosensitizers have been introduced in the RT procedure with enormous potential in cancer therapy^6^. The radiosensitizing agents usually trigger tumor cells to be more sensitive to ionizing radiation by promoting the generation of free radicals and accelerating DNA damage. Recently, there has been rapid progress in the development of nanotechnology-based materials in combination with new medical technologies, which have provided promising and rational design in the development of radiation sensitizers with excellent physicochemical features such as intrinsic radiosensitivity, high drug loading capacity, capability of tumor-targeting by enhanced permeability retention (EPR) effect, appropriate biocompatibility and low toxicity to normal tissues^7–9^. Nanotechnology and the use of nanoparticles (NPs) based on metal atoms with high atomic numbers in combination with radiation therapy to enhance the effect of radiation by accumulating more in the tumor area compared to healthy tissues can effectively increase the efficiency of radiation therapy and reduce side effects^10,11^. NPs of high atomic number elements such as gold, bismuth, platinum, silver and some lanthanides can increase the effective dose of ionizing radiation in tumor tissue and consequently the therapeutic effect due to their high ionizing radiation absorption mechanism and emissions of secondary electrons with Compton scattering and photoelectric effect^12^. The presence of NPs in cancer cells increases the production of ROS, which increases oxidative stress and the amount of effective radiation damage by restricting the cells in the radiation-sensitive phase (M, G2) and inhibiting DNA repair^13–15^.

Silver NPs (AgNPs) are virtually non-toxic at low concentrations, but they can accumulate in mammalian cells and interact with intracellular components, causing side effects and infections in various organs. The properties of AgNPs strongly depend on their size, shape and crystallinity. Their large specific surface area and high surface energy lead AgNPs to self-aggregate, which significantly reduces their application. Silver NPs are used for multifunctional biomedical applications^16,17^. It is beneficial to utilize different stabilizers to solve this limitation^18,19^. Researchers have recently extensively used albumin protein to coat and stabilize various NPs such as gold, silver and iron oxide NPs^20,21^. Bovine serum albumin (BSA) has found many applications in drug delivery due to its easy production and reasonable price, high drug loading capacity, convenient targeting, ligand binding and cell uptake capabilities, biocompatibility and biodegradability^22^. For instance, in 2013, US researchers designed BSA-coated AgNPs with an FCC crystal structure and an average size of 11–15 nm. They used BSA as an effective protective agent to prevent the accumulation of AgNPs. Thiol-containing cysteine residues bonded with AgNPs, possibly via direct chemical interactions, generating a stabilizing effect due to the presence of bulky protein molecules. The findings also showed that the composition and structure of AgNPs/BSA can have a major impact on the bioavailability, antimicrobial activity and overall behavior of protein-conjugated NPs in the biological system^21^. In addition, De-Hao et al. showed that the colloidal stability of BSA-conjugated AgNPs increase significantly compared to bare NPs over the pH range of 2.3–7^23^. Peidang et.al also introduced AS1411 aptamer and verapamil conjugated BSA coated AgNPs (AgNPs@BSA-AS-VRP) as promising radiosensitizing and radioresistance agent for glioma radiotherapy. Their results yielded a sensitization enhancement ratio (SER) of 1.55, suggesting excellent potential for enhancing the efficacy of radiotherapy^24^.

**Figure 1:**
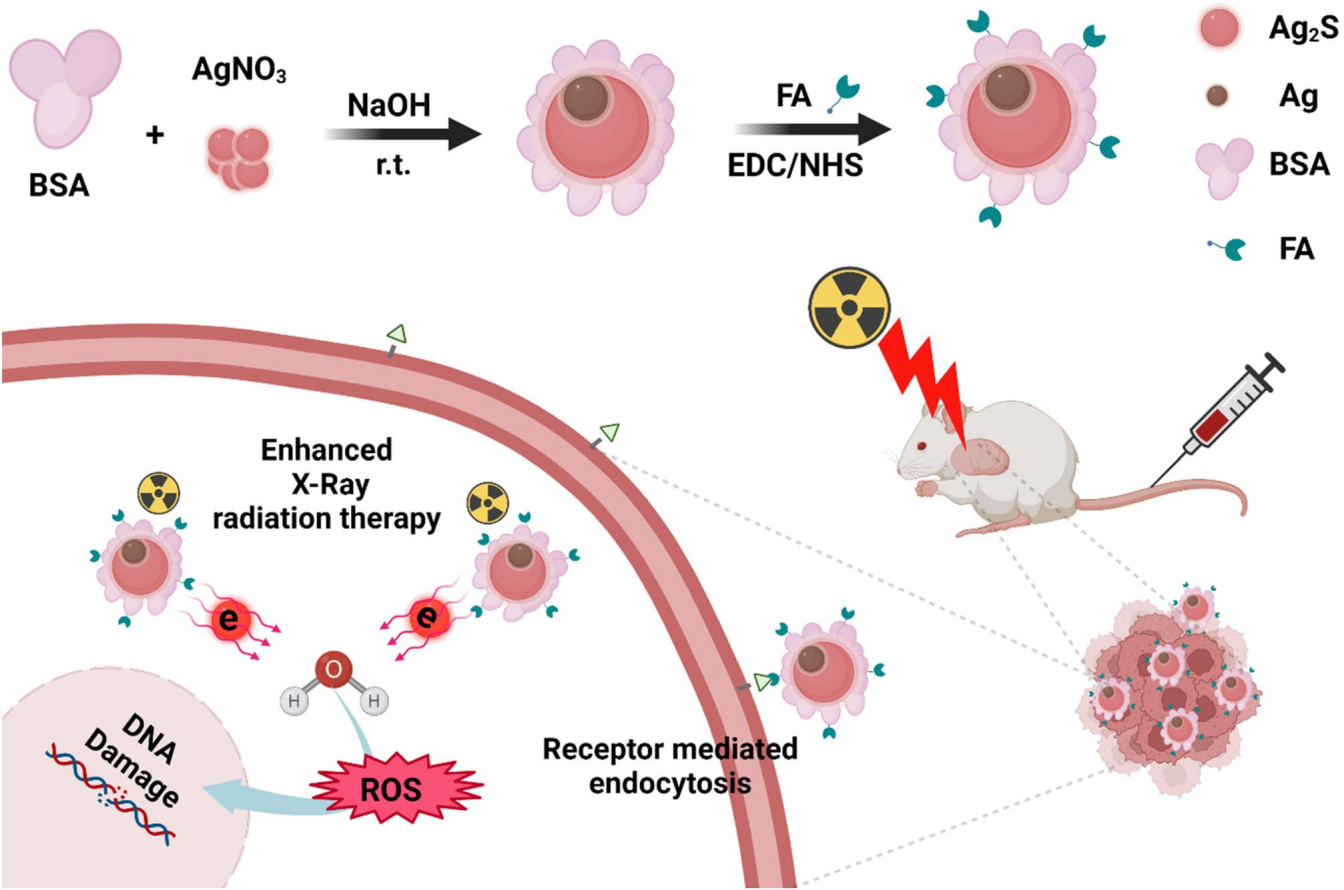
Schematic illustration of the synthesis of Ag-Ag_2_S@BSA NPs and Ag-Ag_2_S@BSA-FA NPs, and their radiosensitizing function.

It is important to note that using Ag_2_S with Ag NPs can generate NPs whose properties may be markedly different in comparison to Ag_2_S and Ag NPs alone, due to the interactions resulting from small variations in electron transferring across the interface between these two limited electron ‘nano-reservoirs’. Having excellent photoelectric and photocatalytic properties as well as chemical stability^25,26^ of Ag and Ag_2_S nanomaterials, we decided to evaluate the properties of Ag-Ag_2_S Janus NPs as radiosensitizers for radiotherapy (Fig. 1). This study aims to introduce novel Janus nanoradiosensitizers to improve the efficacy of in vitro and in vivo radiotherapy of breast cancer treatment by targeting the tumor via FA conjugated and BSA coated Ag-Ag_2_S NPs to sensitize tumor cells to radiation-induced damage.

## Materials and methods

All methods used in this study were conducted in accordance with relevant guidelines and regulations of the Ethics Committee of the Iran National Science Foundation (Code: INSF-98026551).

### Materials

Silver nitrate (Ag(NO_3_).5H_2_O), bovine serum albumin (BSA), folic acid (FA), N-(3-Dimethylaminopropyl)-N′-ethylcarbodiimide hydrochloride (EDC), N-Hydroxysuccinimide (NHS), dimethyl sulfoxide (DMSO) and 3-(4,5-Dimethylthiazol-2-yl)-2,5-Diphenyltetrazolium Bromide) (MTT) were purchased from Sigma-Aldrich (St. Louis, USA). Nitric acid (HNO_3_), sodium hydroxide (NaOH) and ammonium hydroxide (NH_4_OH) were purchased from Merck (Kenilworth, USA) and used without any further purification. All glassware and magnetic Teflon coated beads were cleaned in the aqua regia solution, then repeatedly washed with distilled water (DI) and dried in the oven. Other chemical materials were provided by a local market.

### Methods

#### Synthesis of silver-silver sulfide NPs coated with bovine serum albumin (Ag-Ag_2_S@BSA)

125 mg Ag(NO_3_).5H_2_O (2 mL) was slowly added to a 16 mL aqueous solution of BSA (31.25 mg/mL) under stirring. Then  NaOH solution (1.5 M) was added to adjust pH to 12^27^. The reaction was stirred at room temperature with a magnetic stirrer for 24 h. For purification, the reaction solution was dialyzed for 48 h, then stored at 4 °C. The general synthesis scheme of Ag-Ag_2_S@BSA NPs is illustrated in Fig. 1.

#### Synthesis of FA conjugated Ag-Ag_2_S@BSA (Ag-Ag_2_S@BSA-FA)

For binding FA to the surface of Ag-Ag_2_S@BSA NPs, the carboxyl groups of FA were first activated by EDC/NHS. For this purpose, 31.6 mg of EDC, 20 mg of NHS and 15 mg of FA were dissolved in 2 mL of DMSO. The above solution was then added to an aqueous solution containing 150 mg of Ag-Ag_2_S@BSA NPs. After adjusting the pH of the reaction solution to 8.2 with NaOH, the solution was stirred in a dark environment for 24 h. To remove unreacted FA and other reactants, the reaction solution was dialyzed. The general scheme of Ag-Ag_2_S@BSA-FA synthesis is shown in Fig. 1.

#### Preparation of fluorescein isothiocyanate (FITC) labeled Ag-Ag_2_S@BSA-FA

To covalently attach FITC moieties to the surface of the NPs through the reaction of isothiocyanate groups of FITC, a solution of Ag-Ag_2_S@BSA or Ag-Ag_2_S@BSA-FA was mixed with FITC solution in a 1:10 ratio and shaken at room temperature overnight, according to the method in the literature^28^. Then, excess FITC was extracted from the solution by dialysis for 48 h. All steps were performed in dark environment.

### Characterization

The crystalline structure was characterized by X-ray diffraction (XRD; a Bruker AXS model D8 Advance diffractometer) by using a Cu-Kα1 radiation source, λ = 0.15406 nm. The XRD data in 2θ ranging from 10° to 80° were collected with a scanning step size of 0.02°. X-ray photoelectron spectroscopy (XPS) measurements were performed using a mono-chromatized Al K α X-ray source (Thermo Scientific). Morphologies and size of nanostructures were determined by transitions electron microscopy (TEM; FEI 120 kV), and scanning electron microscopy (SEM; Vega Tescan). Dynamic light scattering (DLS; Malvern Instruments, Worcestershire, UK, model Nano ZS) was used to determine the hydrodynamic size of NPs. NPs were dispersed in deionized H_2_O and the size was measured by DLS (Malvern Instruments, Worcestershire, UK, model Nano ZS). Spectroscopic characterizations were carried out using ultraviolet–visible spectroscopy (UV–Vis; Thermo Fisher Scientific, USA, Madison, model GENESYSTM 10S). For analyzing samples by UV–Vis, they were dispersed in deionized H_2_O, and measured in the range of 600–200 nm.

### Colloidal stability of Ag-Ag_2_S@BSA NPs

To monitor the stability of NPs, it was dispersed in water and PBS, then the size of NPs was measured at different time intervals.

### Determination of the amount of FA conjugation

0.5 mg of Ag-Ag_2_S@BSA-FA and proteinase K (1 mL, 65:35 PBS:Ethanol at pH 7) were mixed and shacked at 37 °C for 24 h. Following centrifugation at 18,000 rpm, the supernatant (released FA) was measured with a UV–Vis spectrophotometer at 352 nm to calculate the amount of conjugated FA.

### Cytotoxicity study

To determine the biocompatibility of the prepared Ag-Ag_2_S@BSA-FA, cell viability was evaluated on HFF-2 fibroblast cells by MTT assay. Briefly, HFF-2 cells in 100 μl complete culture medium were transferred to a 96-well plate and incubated at 37 °C with moisture containing 5% CO_2_ for 24 h. The culture medium was considered as the control. The cells were then treated with Ag-Ag_2_S@BSA-FA NPs at three concentrations (1.3, 4 and 12 μg/ml) for 5 h. The culture medium was drained and fresh culture medium was added to each well. In the next step, the cells were incubated for another 24 h. Then, 20 μl of MTT at a concentration of 5 mg/mL was added to each well and incubated for 4 h. Following removing the solution from each well, 100 μl of DMSO was added and then the absorbance of each well was measured at 570/630 nm with a microplate reader (Tecan).

### Hemolysis

Since NPs encounter blood in the biological environment when injected intravenously, it is important to determine their blood compatibility. For this purpose, 500 μl of solution containing NPs in 3 different concentrations and human red blood cells were poured into Eppendorf and placed in a shaker (37 °C) for 4h. It is noteworthy that deionized water was considered as the positive control and PBS as the negative control. The samples were then centrifuged at 13,000 rpm for 15 min and finally the absorbance of the supernatant was measured at 540 nm. The amount of hemolysis was calculated using the following equation.

$$\% {\text{ Hemolysis }} = \frac{{A \left( {sample} \right) - A \left( {negative} \right)}}{{A \left( {positive} \right) - A \left( {negative} \right)}} \times 100$$

### LD50 assay

The LD50 test was used to determine the toxicity of NPs upon injection into the Balb/C mice. Different doses of 0, 44.44, 66.66 and 100 mg/kg were intravenously injected into mice (N = 4) and survival of the mice was monitored for 30 days.

### Cytotoxicity study on cancer cells

MTT assay was used to determine the radiosensitizing ability of NPs under X-ray irradiation. The mouse breast cancer cells (4T1) were co-incubated with different NP concentrations. After the 5h incubation period, the medium containing NPs were replaced with fresh culture medium. Following X-ray irradiation of cells at a dose of 4 Gy (6 MV) and incubation for a further 24 h, the above-mentioned MTT assay was performed to determine the cell viability of each group.

### Cellular uptake and internalization efficacy

To determine the effect of the presence of FA moieties on the internalization of NPs into cells, the cellular uptake rate was evaluated by flow cytometry. First, 50,000 cells were placed in a 24-well plate mixed with 500 μl of culture medium, and after 24 h the cells were washed with PBS and co-incubated with FITC labeled NPs (Ag-Ag_2_S@ BSA-FITC and Ag-Ag_2_S@BSA-FA-FITC) at concentrations of 1.3, 4 and 12 μg/mL. After 5 h, the samples were washed with PBS, then trypsinized and analyzed by flow cytometer (BD Biosciences, San Jose, CA).

### Intracellular ROS generation

To determine the ability of designed NPs to produce ROS, the 4T1 cells were cultured in a 96-well plate with 5000 cells and incubated overnight. After incubation, cells were co-incubated with different treatment groups, including control, X-ray, Ag-Ag_2_S@BSA-FA, Ag-Ag_2_S@BSA + X-ray and Ag-Ag_2_S@BSA-FA + X-ray, for 5 h. Then, cells were washed and incubated for 1 h in RPMI-1640 medium containing 20 µM 2,7-dichlorodihydrofluorescein diacetate (DCFH-DA). In the next step, cell wells were irradiated (4 Gy, 6 MV) and observed by a fluorescence microscope.

### Calcein AM/PI cell staining assay

Nanoradiosensitizer-triggered death of 4T1 cells upon X-ray irradiation was exploited via the Calcein AM/PI (propidium iodide) staining method. Briefly, 4T1 cells were seeded in a 96-well plate (5000 cells per well) and incubated for 24 h, followed by exposure to various treatment regimens, including control, X-ray, Ag-Ag_2_S@BSA-FA, Ag-Ag_2_S@BSA + X-ray and Ag-Ag_2_S@BSA-FA + X-ray, they were incubated for another 24 h. Next, Calcein AM (3 μM) was added and the cells were incubated for 30 min followed by 5 min incubation with PI (4 μM). Eventually, green (live) and red (dead) fluorescence images of 4T1 cells were documented.

### Clonogenic assay

4T1 cells were seeded in a 6-well plate with complete medium cell culture and incubated for 24 h. The intended samples were then added to the wells, which were either exposed or not by X-ray. Then, the cells were incubated for an additional 7 days, fixed with methanol:acetic acid (3:1) and stained with crystal violet. Finally, the number of colonies was counted^29^.

### In vivo study

10^6^ 4T1 cells were injected subcutaneously into the right flank of Balb/C mice in order to generate tumors. After the tumor volume reached 100 mm^3^, the mice were randomly divided into 5 groups (N = 5): PBS, PBS + 4 Gy X-Ray, Ag-Ag_2_S@BSA + 4 Gy X-Ray, Ag-Ag_2_S@BSA-FA and Ag-Ag_2_S@BSA-FA + 4 Gy X-ray. For the treatment plan, targeted mice were exposed to X-Ray (4Gy, 6MV) 24 h after intravenous injection of NPs. They were returned to the animal chamber after irradiation to evaluate the treatment process, tumor size and weight of the mice. The following formula was used to calculate the relative tumor volume.

$${\text{Relative Tumor Volume}} = \frac{{\left( {Tumor\, length} \right) \times (Tumor\, width)^{2} }}{2}$$

### Histology study

The tumors and key organs were harvested from mice in different groups and fixed in a 4% paraformaldehyde solution. The specimens were then sent to a histology laboratory for histopathological evaluation.

### Statistical analysis

All of the quantitative data were expressed as the mean with standard deviation (mean ± SD) unless otherwise stated. Statistical analysis was performed using GraphPad Prism 8 software.

### Ethics approval and consent to participate

This study was approved by the Ethics Committee of the Iran National Science Foundation (Code: INSF-98026551). Experimental descriptions in this manuscript comply with the ARRIVE guidelines.

## Results

### Characterization

**Figure 2:**
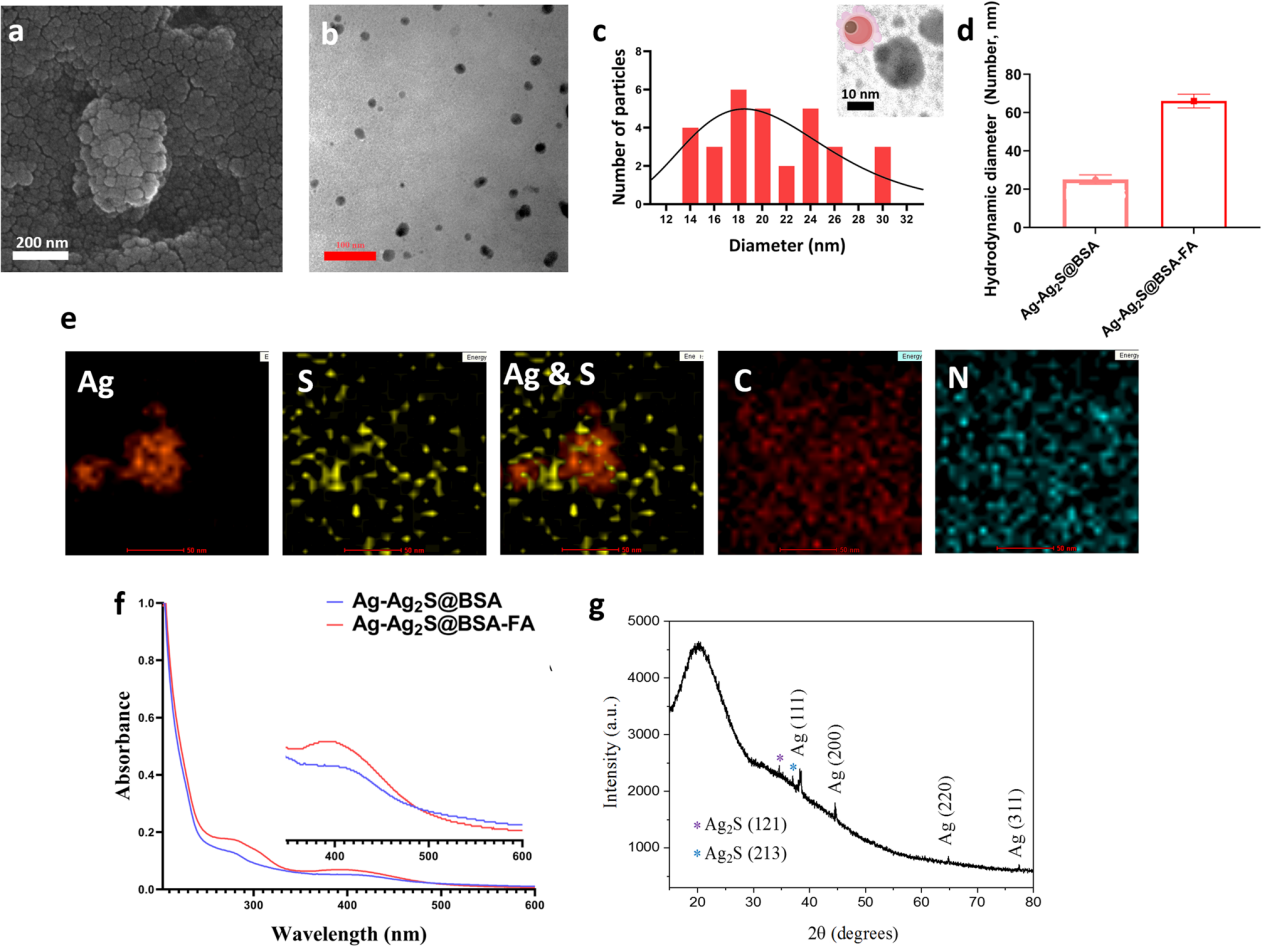
(**a**) SEM; (**b**,**c**) TEM image and related size distribution of Ag-Ag_2_S@BSA-FA (Insert image: TEM image of Ag-Ag_2_S@BSA); (**d)** Hydrodynamic size of Ag-Ag_2_S@BSA and Ag-Ag_2_S@BSA-FA; (**e**) TEM-EDS-mapping of Ag-Ag_2_S@BSA-FA NPs; (**f**) UV–Vis spectra of Ag-Ag_2_S@BSA and Ag-Ag_2_S@BSA-FA NPs; (**g**) X- ray diffraction pattern of Ag-Ag_2_S@BSA NPs.

Here, we synthesized promising novel radiosensitizers based on Janus-type Ag-Ag_2_S NPs for enhanced breast cancer treatment in a murine model. Ag-Ag_2_S@BSA NPs were prepared by a well-documented biomineralization process in a basic media^30^. It is well-known that BSA may be denatured to liberate several residues under strong basic conditions^31,32^. Under high basic conditions, BSA may be denatured to release several residues. For example, cysteine is a great sulfur source for producing metal sulfide NPs^27,33^. In this nature-inspired process, BSA acts both as a sulfur source for the preparation of Ag_2_S and as a stabilizer and scaffold in the mineralization of Ag-Ag_2_S nanocrystals, similar to the production of inorganic biominerals through the regulation of biological macromolecules in living organisms. Following their synthesis and subsequent modifications with targeting agent, Ag-Ag_2_S@BSA-FA NPs were characterized by several methods. As can be seen from the SEM image of Ag-Ag_2_S@BSA-FA NPs taken from the powder sample (Fig. 2a), the NPs are obscured by BSA, but they are still almost completely spherical and can be seen individually. From the TEM image taken after the powder was dissolved in water (Fig. 2b), it can be seen that Ag-Ag_2_S@BSA-FA NPs are well dispersed without aggregation, spherical with a size of approximately 20.60 ± 4.7 nm (n = 30), and have a uniform size distribution. As can obviously be seen in the inserted image of Fig. 2c small Ag NPs appear to be in close contact with Ag_2_S spheres. As a result of different phase compositions and electron densities, different contrasts are seen in the TEM image, confirming the existence of two different NPs. Although there is no discernible interface, small Ag NPs appear to be in close contact with Ag_2_S spheres, like a patch or a matrix (well-defined uniform Janus structures)^26^.

From DLS analysis, Ag-Ag_2_S@BSA NPs presented an average hydrodynamic diameter of 25 nm and a polydispersity index (PDI) of 0.457 (Fig. 2d). Size monitoring of NPs shows that, there was no significant size difference during one months (Fig. S1). After conjugation of FA to the construct, the size slightly increased to 66 nm, while PDI was found to be 0.474 for Ag-Ag_2_S@BSA-FA (Fig. 2d). The DLS technique was also used to assess the surface charge of the final formulation. According to zeta potential measurements performed with DLS, the surface charge of Ag-Ag_2_S@BSA-FA NPs was found to be -23.2 mV, which is quite promising for the reasons mentioned above. DLS result indicates that this system is suitable in size for biological studies, including in vivo assays. The surface charge of the particles is a parameter for the system's colloidal stability. Highly negatively or positively charged NPs tend to repel each other and do not tend to aggregate. NPs interact with or adhere to oppositely charged cell layers, therefore, surface charge is also important in the accumulation of NPs in the bloodstream. Plasma and blood cells (biological elements) have a negative charge. For this reason, negatively charged NPs cannot electrostatically bind to blood cells and thus have a high circulation time in the blood^28,29^. Composition was also confirmed by TEM-EDS mapping which highlighted the presence of Ag, and S elements in the structure of hybrid (Fig. 2e).

A UV–Vis spectrophotometer was used to investigate the presence of the components of the Ag-Ag_2_S@BSA and Ag-Ag_2_S@BSA-FA constructs. As shown in Fig. 2f, the broad peak around 400 nm in the spectra of Ag-Ag_2_S@BSA and Ag-Ag_2_S@BSA-FA NPs suggests the presence of silver NPs. BSA absorbance was seen around 268 nm in both spectra. After folic acid binding to Ag-Ag_2_S@BSA NPs, a new peak around 302 nm appeared, which matched well with FA. The appearance of peaks at 400 nm, 268 nm, and 302 nm corresponded to silver NPs, BSA coating and FA, respectively^34,35^. The amount of FA bound to the surface of Ag-Ag_2_S@BSA was measured at 352 nm after its release using proteinase K, resulting in a loading rate of ~ 8.2%.

The X-ray diffraction pattern of Ag-Ag_2_S@BSA NPs in Fig. 2g presents a broad peak centered around 2θ of 20° corresponding to the BSA^26,28^. The XRD peaks observed at 2θ of 38.2°, 44.3°, 64.7°, and 77.4° can be attributed to the (111), (200), (220) and (311) crystallographic planes of face-centered cubic silver nanocrystals, respectively, and match well with the JCPDS card, no. 65-8428 for silver NPs^36^. In addition, slight peaks at 34.6° and 37.0° correspond to the (121) and (213) planes of Ag_2_S NPs (JCPDS card, no. 65-2356), respectively^26,37^. Thus, the XRD study confirmed the presence of silver and silver sulfide in Ag-Ag_2_S@BSA and the absence of other obvious phases in the structure as impurities.

**Figure 3:**
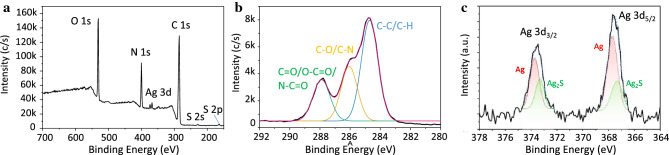
XPS analysis of Ag-Ag_2_S@BSA-FA NPs: (**a**) Wide-scan survey spectrum, (**b**) High-resolution XPS spectrum of C 1s photoelectrons, and (**c**) High-resolution XPS spectrum of Ag 3d photoelectrons.

In order to further elucidate the structure of Ag-Ag_2_S@BSA-FA NPs and to investigate the oxidation state of Ag we have carried out XPS analysis. The wide range XPS scan, depicting the strong existence of binding energies for C 1s, O 1s, N1s, Ag 3d, and S 2p, is shown in Fig. 3a. The high C, O and N content in this spectrum, reaching 61.0%, 17.8% and 17.2%, respectively, indicates that the surface composition of the NPs to a depth of about 10 nm is largely dominated by BSA and FA. The N/O molar ratio detected by XPS is about 0.97. This ratio implies a higher nitrogen content in comparison to the chemical composition of BSA. This elevated nitrogen presence can be attributed to the integration of nitrogen-rich FA into the structural framework. The C1s core-level scan in Fig. 3b confirms the presence of BSA and FA by showing the characteristic C–C/C–H, C–O/C–N and C=O/O–C–O/N–C=O components consistent with their structures^38,39^. The Ag 3d spectrum is characterized by the doublets arising from spin orbital separation, which correspond to the core levels of Ag 3d_5/2_ and Ag 3d_3/2_ at approximately 367.7 and 373.6 eV, respectively^40,41^. The higher binding energy components can be assigned to zero-valent species of Ag NPs, while the peaks at about 367.4 eV and 373.3 eV may be attributed to Ag(I) ions in Ag_2_S (Fig. 3c)^42^, in the structure of Ag-Ag_2_S@BSA-FA NPs. The binding energies for Ag (0) and Ag (I) species overlap due to very similar peak positions. However, it is reported that Ag 3d photoelectrons shift slightly to lower binding energies when Ag–S bond is formed^19,43–45^. As can be seen in Fig. 3c, Ag 3d_3/2_ and Ag 3d_5/2_ core levels are clearly curve‐fitted into two Ag and Ag_2_S peak components, confirming the coexistence of both nanocrystals in Ag-Ag_2_S@BSA-FA NPs.

### In vitro assays

#### Analysis of hemocompatibility

**Figure 4:**
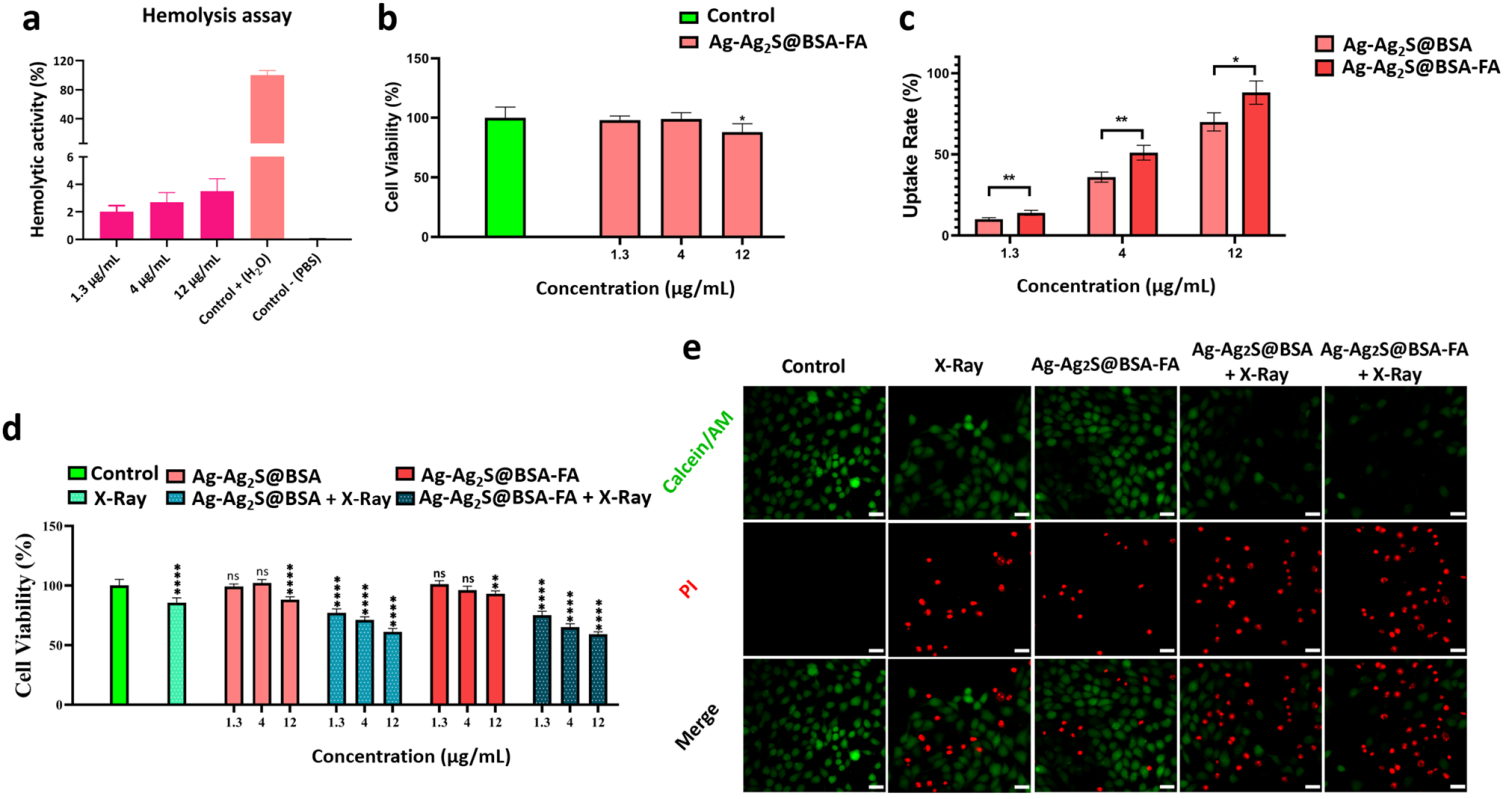
(**a**) Percentage of hemolysis of Ag-Ag_2_S@BSA-FA NPs in different concentrations; (**b**) Cytotoxicity of Ag-Ag_2_S@BSA-FA NPs on healthy HFF2 cells; (**c**) Uptake rate of Ag-Ag_2_S@BSA-FA-FITC and Ag-Ag_2_S@BSA-FITC NPs by 4T1 cells analyzed by flow cytometry; (**d**) Cell viability of 4T1 cells under different treatment procedures in the presence and absence of X-ray irradiation (4 Gy) and NPs; (**e**) 4T1 cells images stained with calcein-AM and PI in the presence and absence of X-ray radiation after different treatments. (Scale bar is 20 µm). Data = mean ± SD; Sign “ns” *, **, ***, and **** show no significant difference, and significant difference with p ≤ 0.05, p ˂ 0.01, p ˂ 0.001 and p ˂ 0.0001, respectively, compared to the control group.

To assess the blood compatibility of Ag-Ag_2_S@BSA-FA NPs, hemolysis testing was performed at three concentrations (1.3, 4, and 12 μg/mL) as shown in Fig. 4a. Water was used as a positive control and PBS solution was employed as a negative control. As can be seen in Fig. 4a, the aforementioned samples have a lysis rate of less than 3% even at the highest concentration (12 μg/mL), which indicates an appropriate hemocompatibility. Materials with less than 5% hemolysis are considered non-hemolytic materials based on American Society for Testing and Materials (ASTM)^46, 47^.

#### Analysis of cytotoxicity against healthy cells

MTT assay was used to investigate the cytotoxicity of Ag-Ag_2_S@BSA-FA NPs on healthy HFF2 cells. As shown in Fig. 4b, the results of the MTT assay showed that Ag-Ag_2_S@BSA-FA NPs prepared at concentrations of 1.3, 4 and 12 µg/mL had no noticeable inhibitory effect on the treated cells, which means that the designed NPs were fortunately non-toxic to healthy cells.

#### Cellular uptake and internalization efficacy

As can be seen from Fig. 4c, the cellular uptake results of NPs by the mouse breast cancer cells (4T1) showed a concentration-dependent cell internalization. Moreover, FA-conjugated NPs exhibited a higher uptake rate at all studied concentrations compared to Ag-Ag_2_S@BSA NPs. Due to the overexpression of folate receptors on cancer cells, FA-conjugated NPs are more likely to be internalized by them^48^.

#### In vitro antitumor activity

The MTT assay was used to determine the toxicity of Ag-Ag_2_S@BSA and Ag-Ag_2_S@BSA-FA NPs on the mouse breast cancer cell line (4T1) at three concentrations of 1.3 µg/mL, 4 µg/mL, and 12 µg/mL, in the presence and absence of X-ray radiation. As shown in Fig. 4d, the NPs-free group (containing culture medium only) has 100% viability. As shown in anticancer study result, the therapeutic effect increased with increasing concentrations of NPs. The cell group irradiated by 4Gy dose showed 85% viability. The cell survival rate of the group treated with Ag-Ag_2_S@BSA NPs (1.3 μg/mL) in the presence of irradiation was approximately 77%. The viability of cells co-treated with NPs (12 μg/mL) and X-ray irradiation decreased to 61% and 58% in the presence of Ag-Ag_2_S@BSA and Ag-Ag_2_S@BSA-FA NPs, respectively. These results proved that the prepared NPs exhibited a better therapeutic effect compared to the group that received only X-Ray radiation, suggesting enhanced radiosensitivity in the case of the NPs. The viability of cells irradiated by X-rays exhibited the greatest reduction in the presence of Ag-Ag_2_S@BSA-FA NPs in comparison to other counterparts.

#### Calcein-AM/PI cell staining assay

Viability staining is an important method for determining cell viability in the context of potential therapeutic applications of prepared NPs. As shown in Fig. 4e, the anticancer activity of Ag-Ag_2_S@BSA and Ag-Ag_2_S@BSA-FA under X-ray irradiation were further examined by double-labeling technique using the fluorescent markers Calcein-AM and PI, which stain live and dead cells in green and red, respectively. In Fig. 4e, it is clearly seen that all cells in the control group are alive, there are no dead cells. However, the rate of dead cells gradually increased in Ag-Ag_2_S@BSA-FA, Ag-Ag_2_S@BSA + X-ray and Ag-Ag_2_S@BSA-FA + X-ray groups shown from left to right. Although cell death was observed in all three groups containing silver-based NPs, the Ag-Ag_2_S@BSA-FA + X-ray group with appropriate active targeting led to the observation of the highest proportion of dead cells compared to living ones, as the most effective treatment procedure. Intracellular esterases in living cells can convert the virtually nonfluorescent cell-permeable Calcein-AM into the intensely fluorescent calcein. PI enters cells with damaged membranes and enhances fluorescence by binding to DNA, thereby producing a bright red fluorescence in dead cells^49^. Calcein-AM/PI cell staining assay result is well matched with MTT assay result. Under ionizing radiation such as X-Ray, radiosensitizers could enhance the radiosensitivity of cancer cells^50,51^. Ag NPs demonstrated radio enhancement ability in breast cancer tumor cells^52^. But their ability to efficiently enter and accumulate in tumor cells remains to be improved. It can be improved by active targeting agents. Ag-Ag_2_S@BSA-FA + X-ray group showed a higher cell death rate compared to other groups.

#### Intracellular ROS generation

**Figure 5:**
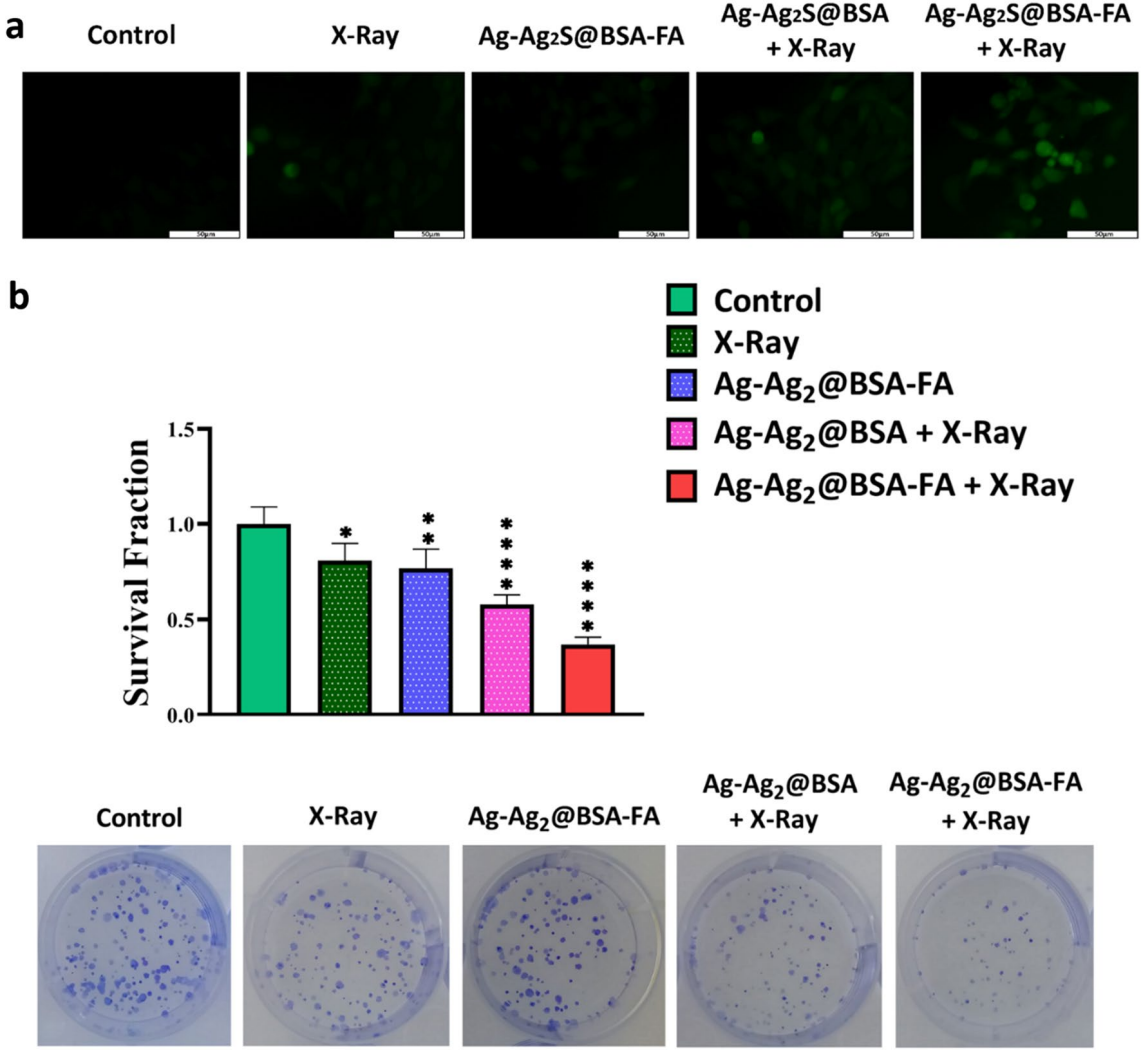
(**a**) Image of 4T1 cells after incubation with intracellular ROS production detector (DCFH-DA); (**b**) Clonogenic assay digital images and survival fraction for Ag-Ag_2_S@ BSA and Ag-Ag_2_S@BSA-FA NPs in the presence and absence of X-ray irradiation. Data = mean ± SD; Sign “ns” *, **, ***, and **** show no significant difference, and significant difference with p ≤ 0.05, p ˂ 0.01, p ˂ 0.001 and p ˂ 0.0001, respectively, compared to the control group.

The DCFH-DA fluorescent probe was used to confirm ROS production within cells. As seen in Fig. 5a, the presence of green color is a criterion of ROS production in the cell. There was no green fluorescence in the control group. However, it was slightly found in the Ag-Ag_2_S@BSA-FA and X-ray groups. When cells were treated with Ag-Ag_2_S@BSA and Ag-Ag_2_S@BSA-FA under X-ray radiation, the florescent intensity increased further, indicating an appropriate production of ROS in these two groups. ROS can damage biomolecules within cells and cause cell death. That’s why it has a key role in RT^50^. The Ag-Ag_2_S@BSA-FA dramatically induce intracellular ROS overproduction under X-ray radiation.

#### In vitro colony formation

A clonogenic assay is an *in vitro* assay based on the ability of a single cell to grow into a colony^53^. The clonogenic assay can be used not only to determine cell death after ionizing radiation therapy (here X- ray), but also to understand the efficacy of other cytotoxic agents. This assay examines the ability of each cell to perform "infinite" division while growing its population. Only a fraction of cells can maintain the colony behavior after implantation in the cell plate. Colony population changes before and after treatment are a criterion for evaluating the test on the cells within 1–3 weeks. In this assay, the cells were stained with crystal violet (0.5%) after implantation, treatment and incubation for one week^53^. As shown in Fig. 5b, the survival fraction of Ag-Ag_2_S@BSA and Ag-Ag_2_S@BSA-FA in the presence of X-ray was halved in comparison to the control group, proving the effectiveness of the successfully designed NPs in radiotherapy treatment. Ag NPs under X-ray irradiation strongly decrease the formation of colonies. The better radiosensitizing activity of Ag-Ag_2_S@BSA-FA NPs may be due to their higher intracellular accumulation, since the radiosensitization effects are directly related to the amount of intracellular radiosensitizers^54^.

### In vivo assays

#### LD_50_ as an in vivo biosafety indicator

**Figure 6:**
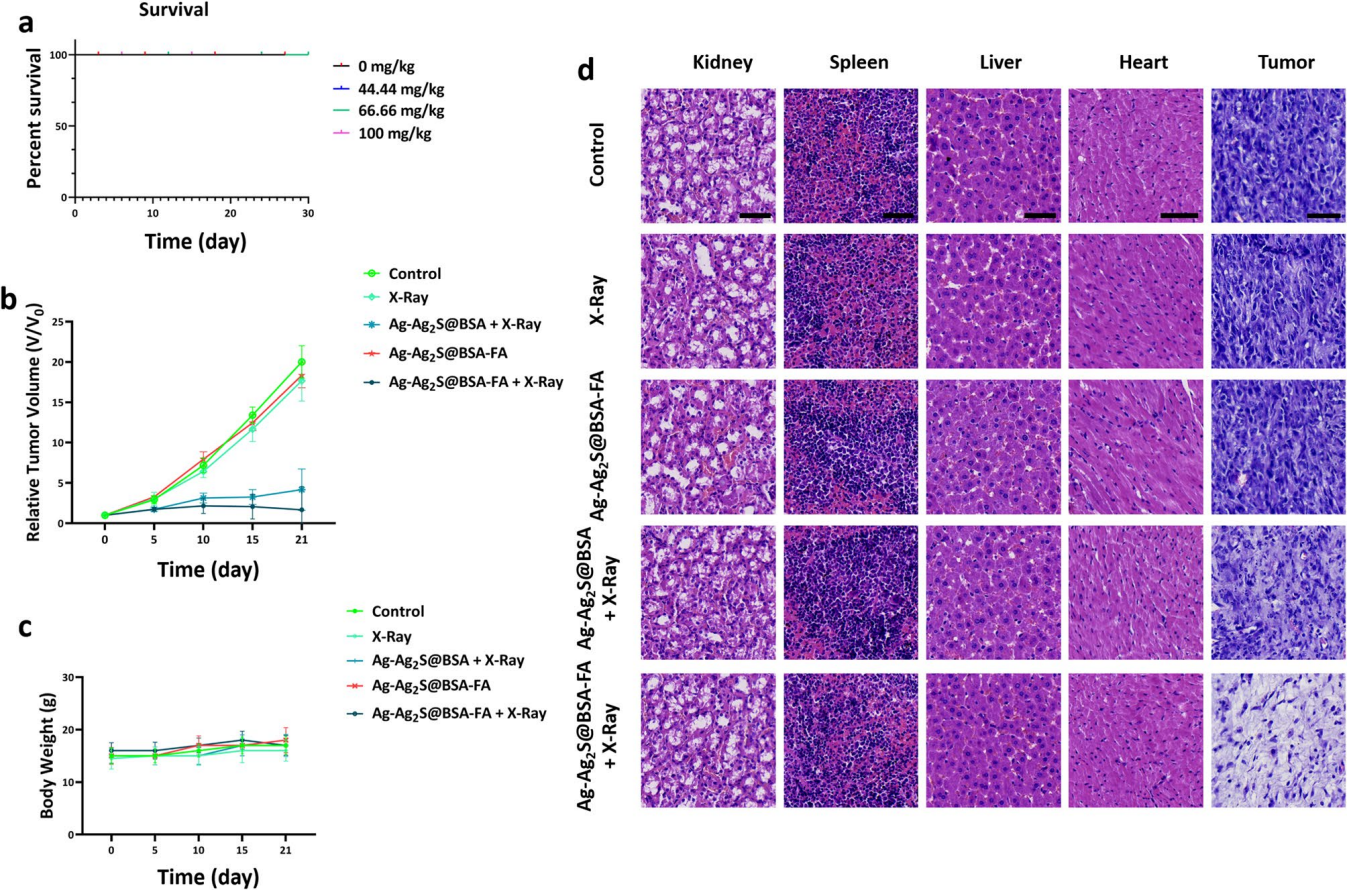
(**a**) Survival of Balb/C mice in LD50 test; (**b**) Relative tumor volume over the treatment period (21 days); (**c**) Weight of mice during treatment; (**d**) Histopathological images of tumors and key organs of mice stained with H&E (scale bar 50 µm).

In addition to determining the toxicity of NPs in in vitro studies by HFF2 cells, LD50 testing was carried out after intravenous injection into Balb/C mice. In more detail, four doses of Ag-Ag_2_S@BSA-FA NPs (0, 44.44, 66.66 and 100 mg/kg) dispersed in phosphate buffer (PBS) were injected through the tail vein of mice. As shown in Fig. 6a by the Cox regression diagram, no mortality was observed for 30 days.

#### In vivo radioenhancing ability

The therapeutic effect of radiotherapy was investigated in vivo in the presence of Ag-Ag_2_S@BSA and Ag-Ag_2_S@BSA-FA NPs. First, 4T1 cancer cells (1 × 10^6^) were injected into the left flank of the mouse to create a tumor. After 12–15 days, when the tumor volume reached 100 mm^3^, treatment procedure was commenced. As shown in Fig. 6b, the control group along with the X-ray and Ag-Ag_2_S@BSA-FA groups had no significant inhibitory effect on tumor growth. However, the inhibitory effect of tumor growth was clearly seen in the groups treated with Ag-Ag_2_S@BSA and Ag-Ag_2_S@BSA-FA NPs under X-ray radiation. Notably, in the Ag-Ag_2_S@BSA-FA group, the tumor volume reached zero by X-ray. Results also showed that Ag-Ag_2_S@BSA-FA NPs had better radiation sensitization than Ag-Ag_2_S@BSA NPs, maybe because of greater tumor-homing and specificity of Ag-Ag_2_S@BSA-FA NPs. The results showed that the designed NPs play an important role in cancer treatment in animal studies. In addition, as shown in Fig. 6c, there was no discernible pattern in the weight of the mice over the treatment period and the treatment had no negative effects on the weight of the mice.

#### Histopathology analysis

The key organs and tumors were fixed with 4% paraformaldehyde solution and stained with hematoxylin and eosin (H&E) after removal from the body. As can be seen in Fig. 6d, the H&E staining confirmed that treatment by Ag-Ag_2_S@BSA-FA NPs with X-Ray irradiation caused the most damage. As shown in Fig. 6d, the groups treated with the NPs showed no obvious abnormalities. NPs considerably inhibited the growth of tumors under X-ray radiation, without inducing any cytotoxicity on normal tissues. No evident tissue damage could strongly confirm the biocompatibility of the injected NPs^35^.

## Conclusion

In this study, Ag-Ag_2_S@BSA-FA NPs with well-defined uniform Janus structures were synthesized using BSA-guided bio-mineralization method. SEM and TEM revealed the presence of homogenous spherical NPs with an average size of ~ 20 nm. XPS and XRD results showed the coexistence of Ag and Ag_2_S nanocrystals in the designed nanoformulation. In addition, the anti-cancer therapeutic effects of the designed NPs in the presence and absence of X-ray radiation were evaluated by a series of in vitro assays on 4T1 cancer cells. The results showed that X-ray irradiation caused more damage to breast cancer cells when applied in combination with radiosensitizer NPs, and this effect was more pronounced in the presence of Ag-Ag_2_S@BSA-FA NPs. According to the data we obtained from fluorescent microscopy on cancer cells, it was confirmed that Ag-Ag_2_S@BSA and Ag-Ag_2_S@BSA-FA produced higher amounts of ROS under X-ray radiation compared to other groups. Finally, similar to in vitro studies, in vivo studies showed that Ag-Ag_2_S@BSA-FA performed best in minimizing tumor volume in Balb/C mice, proving the success of proper targeting and radiosensitization.

### Supplementary Information

Supplementary Figure 1.

## Data Availability

The datasets generated and/or analysed during the current study are not publicly available due to ongoing studies in his line but are available from the corresponding author on reasonable request.
