# Supplementary material for: Facile preparation of silver based radiosensitizers via biomineralization method for enhanced in vivo breast cancer radiotherapy
Source: Sci Rep. 2023 Sep 13;13:15131. doi: 10.1038/s41598-023-40763-9 (PMC10499791; doi:10.1038/s41598-023-40763-9)
Supplement: Supplementary file 1 — Supplementary Figure 1. [file 41598_2023_40763_MOESM1_ESM.docx]

**Facile Preparation of Silver Based Radiosensitizers via biomineralization method for Enhanced *In vivo* Breast Cancer Radiotherapy**

Mohammadreza Ghaffarlou^1^, Ali Mohammadi^2^, Navid Mousazadeh^2^, Marziyeh Salehiabar^2^, Yahya Kalantari^2^, Jalil Charmi^2^, Murat Barsbay^1^, Yavuz Nuri Ertas^3,5^, Hossein Danafar^2^, Hamed Rezaeejam^4*^, Hamed Nosrati^2*^, Siamak Javani^6,7*^,

1. Hacettepe University, Department of Chemistry, Beytepe, Ankara 06800, Turkey.
2. Zanjan Pharmaceutical Biotechnology Research Center, Zanjan University of Medical Sciences, Zanjan, Iran.
3. ERNAM—Nanotechnology Research and Application Center, Erciyes University, Kayseri 38039, Turkey.
4. Department of Radiology, School of Paramedical Sciences, Zanjan University of Medical Sciences, Zanjan, 45139- 56184, Iran.
5. Department of Biomedical Engineering, Erciyes University, Kayseri 38039, Turkey.
6. Medical Cellular and Molecular Research Center, Golestan University of Medical Sciences, Gorgan, Iran
7. School of Advanced Technologies in Medicine, Golestan University of Medical Sciences, Gorgan, Iran

*Corresponding Authors:

[hrezaeejam@yahoo.com](mailto:hrezaeejam@yahoo.com) (H. Rezaeejam), Nosrati.hamed2020@gmail.com (H. Nosrati), and siamackjavani@yahoo.com (S. Javani)


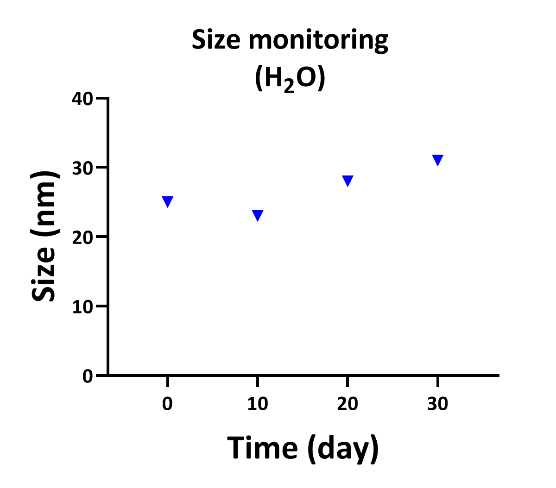

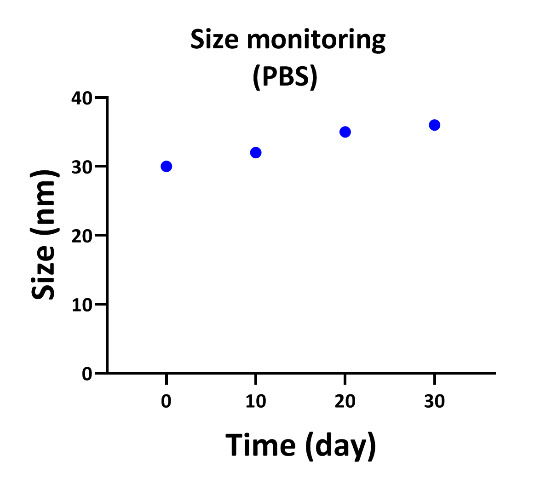


Figure S1. Hydrodynamic size of NPs to screen colloidal stability of solutions containing Ag-Ag2S NPs in water and PBS.
